# Registries: An essential tool for maximising the health benefits of immunisation in the 21st century

**DOI:** 10.2807/1560-7917.ES.2017.22.17.30523

**Published:** 2017-04-27

**Authors:** N S Crowcroft, D Levy-Bruhl

**Affiliations:** 1 Chief, Applied Immunization Research and Evaluation, Public Health Ontario, Toronto, Ontario, Canada; 2 University of Toronto, Toronto, Ontario, Canada; 3 Santé publique France, Saint-Maurice, France

**Keywords:** Vaccines and immunisation Research, evaluation immunisation coverage, vaccination registries, immunisation information systems, vaccines

The variety of available vaccines and the intricacy of immunisation schedules has increased progressively over recent decades. Consequently immunisation programmes have become more complex with the addition of different vaccines such as those against *Haemophilus influenzae* type b infections, meningococcal and pneumococcal disease, human papilloma virus, rotavirus, varicella and herpes zoster to vaccination programmes, and their extension to cover the whole life course. At the same time, the survival of people with chronic and immuncompromising conditions with higher susceptibility to infectious diseases and needing specialist advice on vaccine indications and contra-indications has also increased. It is therefore not surprising that the need has also grown for better data on when, where and who received which vaccine.

High income countries have reached the point where such data are an essential part of any immunisation programme. While clinicians need good information on the protection of their patients to ensure high standard of care, the individual citizen expects to be able to access their own records as well and public health authorities need to be able to identify and respond quickly to concerns in order to maintain the confidence of an increasingly vaccine hesitant public. Immunisation registries have great potential to be the most robust and systematic approach to providing data on the safety and effectiveness of immunisation programmes as well as information whether they reach their target communities and birth cohorts. They hold the information needed for rapid response as well as for the longer term, and can safeguard the immunisation records of individuals over their lifetime.

Many countries across the globe are working towards developing immunisation registries [1] and can usefully share many lessons along the road [2]. In that context, the collection of articles in this issue of *Eurosurveillance*, which follows on an earlier special issue on the topic in 2012 [3], illustrate the evolution and potential of immunisation registries to impact on health. Examples are provided on how an Immunisation Information System (IIS) enables better management of programmes as well as research and evaluation, all of which leads to quality improvement and innovation. Although every registry may have to be different in order to adapt to local particularities in immunisation programme recommendations, legal context, data availability and healthcare delivery systems, those working in the field can still learn much from each other [4]. For example, while countries such as those in northern Europe have data systems integrated through a unique personal identification number, countries lacking this capacity may be able to create similar functionality through data linkage. Furthermore, many requirements, processes and principles are shared. For example ‘No duplicate entry or collection’ is an excellent principle that underpins the system design in Finland as shown by Baum et al. [5].

Altogether, the results of a survey conducted by the European Centre for Disease Prevention and Control (ECDC) presented by Derrough et al. show a positive trend in the implementation of vaccination registries within European countries [4]. Of 27 responding countries of the European Union/European Economic Area, 21 answered that they have an immunisation registry in operation or being currently piloted, either at national or subnational level. Furthermore, of the six remaining countries, four mentioned that they have concrete plans to implement one in the near future. By comparison, in a survey conducted by the Vaccine European New Integrated Collaboration Effort (VENICE) I project in 2007, only 15 of the 27 responding countries had either a national or regional computerised immunisation registry [6].

Two features can be considered as essential for ensuring reliable coverage estimates; the possibility to capture vaccines administered in the past, mentioned by 14 of 16 countries, and those administered outside the public system, which is not the case in many surveyed countries. The impact will of course depend on the contribution of the private sector to vaccination coverage, information not available in the survey.

Other characteristics of registries for example, the functionality for vaccine providers to identify unvaccinated patients and the system to send vaccination reminders, access to the system by vaccine providers and the general public are very heterogeneous. The extent to which a registry can increase vaccine coverage (not only monitor it) and engage both health professionals and the public in taking a proactive role with respect to vaccination depend greatly on these characteristics.

Few countries mentioned the use of their registry for vaccine effectiveness studies. However, 13 of 14 countries mention the possibility of database linkage which would enable this use to increase in future.

The next step, as suggested by the authors, is to bring together key stakeholders involved in countries’ e-health and vaccination programme management to work together on common standards and share experience, expertise and tools. ECDC has definitively, among other institutions, the legitimacy to take an important part in catalysing such an important endeavour.

Differences between registries often reflect local enablers and constraints including privacy legislation and may not be a real barrier if the clinical details included at each level in the system are well aligned with that required for adequate analysis and the needs of organisations targeted for action. For example, in larger federal countries personal health information may not be needed for aggregate coverage estimates at national level. Systems need to be designed appropriately to be able to drive action at different levels. A system which is defined by geography only [7] misses the important alignment between a registry and service providers, who may not be geographically defined.

IIS require significant technical expertise as well as resources and dedication to quality improvement. The technical expertise required is often under estimated [8], and is multi-disciplinary, not only in respect to the information technology. One of the hardest elements to track down is often details of past recommendations, leading some of us to keep hold of old immunisation guides long after they are out of date, as they are sometimes the only available historical record (Figure).

**Figure:**
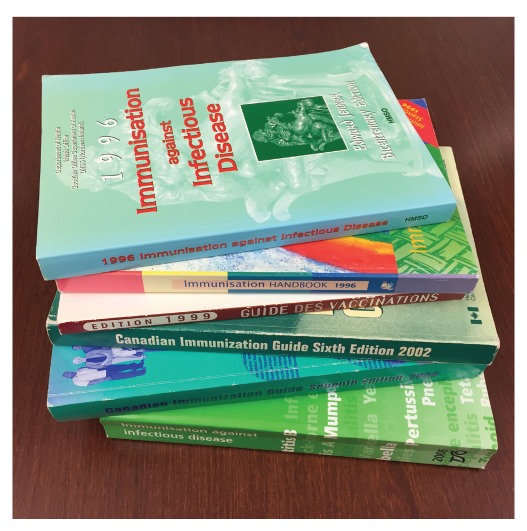
Immunisation guides from various years and countries.

In addition to familiarity with changes that have occurred during the history of the programme and the current recommendations, detailed knowledge is needed of how rules embedded in the registry may affect how immunisation status and coverage is measured. This is essential to understand the implications for identifying unvaccinated individuals and communities. The article from Norway by Hagerup-Jenssen et al. illustrates the technical knowledge and level of responsiveness required to be able to identify and delineate issues in order to improve systems and processes [9].

Good management and attention to detail are essential for the success of all immunisation programmes, when so many elements and actors are involved all the way from the vaccine industry through to the patient. When immunisation programmes are linked to elimination targets, requirements are even more stringent. Growing pressure to deliver on targets to eliminate measles in Europe require measles vaccination coverage to approach levels of nearly 100% in fact, not in fiction [6]. This may require systems with high precision to detect immunisation gaps, given that a critical community size to sustain measles transmission is only a few hundred thousand people [10].

The World Health Organization (WHO) and national reporting requirements focus on individual antigen-specific coverage data e.g. for diphtheria, pertussis, tetanus, measles etc. However, even in a country such as Denmark, where coverage is generally high, it is good to be reminded that a substantial proportion of children may have missed at least one dose of any of their recommended vaccines. Written reminders generated by vaccine registries may not be the whole answer, but seem to be effective in promoting higher coverage [11], particularly in older children in whom missed opportunities may have played a role. Further evaluation of how best to design such communications initiatives in order to increase their impact may be helpful [12]. The Danish study by Suppli et al. also shows how timing is important. Unvaccinated children need to be identified close to the due date so that children are not left unprotected too long, but with enough distance to maximise the effectiveness of reminders. The timeliness of data collection needs to be proportionate and aligned with the effectiveness for action.

Coverage data are a key component of immunisation programme research and evaluation designed to innovate and maximise the benefits of vaccines [13,14]. Such implementation research and evaluation is applied and usually requires a multi-disciplinary approach [15]. If immunisation data are collected within the same information system as morbidity data, or easily linked, this brings additional strengths. The potential is beautifully illustrated by a study from Germany by Rieck et al., demonstrating how the registry can enable vaccine effectiveness assessment, in this case for the varicella vaccine [16].

From a patient engagement perspective, it is heartening to see patient and parent access developing, as well as opportunities for proactive participation, which will be further enhanced by access to data through devices such as mobile telephones [17] and by tailored text messages [18]. In the future, vaccine barcoding will add further functionality and improved data capture to immunisation registries [19]. Functionality can be further enhanced through data linkage, for example to assess equity of access for marginalised groups such as refugees, aboriginal and migrant populations.

Immunisation registries are a long-term commitment, mirroring the fundamental nature of vaccination programmes that protect the population for the whole life course and long-term. Registries should reflect that vision; they should be designed to be sustainable and be seen as an integral part of the immunisation programme that enables maximising their health benefits in multiple ways. The resources required may not be substantial if viewed as part of the total budget for immunisation programmes and an essential intervention, within a broader e-health strategy, that will protect all the members of our communities for the long lives we hope they will lead.
